# camlhmp: a simple framework for building reproducible microbial genome-based typing tools

**DOI:** 10.1128/mra.00201-26

**Published:** 2026-04-20

**Authors:** Robert A. Petit, Chayse M. Rowley, Taylor R. Fearing, Stefaan Verwimp, Rob G. Christensen, Jim A. Mildenberger, Joseph M. Reed, Timothy D. Read

**Affiliations:** 1 Wyoming Public Health Laboratory, Wyoming Department of Health, Cheyenne, Wyoming, USA; 2 Center of Applied Pathogen Epidemiology and Outbreak Response, Georgia PGCoE, Atlanta, Georgia, USA; 3 Computational Systems Biology, Department of Microbial and Molecular Systems, KU Leuven 26657, Leuven, Flanders, Belgium; 4 Division of Infectious Diseases, Department of Medicine, Emory University School of Medicine 12239, Atlanta, Georgia, USA; University of Michigan 1259, Ann Arbor, Michigan, USA

**Keywords:** bioinformatics, software, sequence-based typing, microbial genomics

## Abstract

Sequence-based typing is critical for microbial genomics, yet existing tools are often developed in isolation, leading to duplicated effort, inconsistent formats, and limited community participation. To address this, we developed camlhmp (https://github.com/rpetit3/camlhmp), a framework for creating, executing, and maintaining typing tools with simple, human-readable YAML files.

## ANNOUNCEMENT

In microbiology, genetic typing by PCR-based NAATs (Nucleic Acid Amplification Tests) (1) is commonly used to define genotypes and infer phenotypes. However, with the prevalence of whole-genome sequencing, new bioinformatics-based approaches are increasingly being developed (2). Developing a new sequence-based typing (SBT) tool requires extensive knowledge of the target organism and bioinformatics expertise. Furthermore, a lack of adherence to standard development practices limits community contributions.

Recognizing the need for a standardized, accessible SBT framework, we developed camlhmp (*Classification through yAML Heuristic Mapping Protocol*), a Python-based framework designed to simplify the creation and management of SBT tools. It uses YAML (3), a simple human-readable data serialization language, to define a typing schema. From the schema, camlhmp uses BLAST+ (4) to align target sequences against an input genome to assign types based on the schema-defined heuristics (e.g. exact match, percent coverage thresholds). camlhmp has been developed for x86-64 Linux and macOS systems and requires Python (v3.11+), BLAST+ (v2.15.0+), Biopython (v1.83+) (5), and supporting open-source Python libraries. To ease dependency management, camlhmp is available from Bioconda (6) and PyPi.

The camlhmp (v1.1.4) framework provided a command-line interface (CLI) and an application programming interface (API), which took a user-supplied YAML schema and FASTA sequence file as inputs.

Each YAML schema contained metadata, engine, targets, aliases, and types sections. metadata included fields to describe the schema, such as name and description. engine included a description of the tool used for alignment. targets included a list of all defined targets within the schema, each with a reference sequence. aliases allowed for a name to represent a group of targets. Finally, types defined each type based on the presence of specified targets or aliases. The camlhmp framework includes schema validation functions and example schemas are provided through stand-alone tools (Table 1) and the test suite.

**TABLE 1:** Tools utilizing the camlhmp framework

Tool	Description	Typing approach
pasty	Serogrouping *P. aeruginosa* with O-specific antigen clusters (7)	camlhmp-blast-regions CLI
pbptyper	Typing penicillin-binding protein (PBP) in *S. pneumoniae* (8)	camlhmp API customization
sccmec	Typing SCCmec cassette in *S. aureus* samples (9, 10)	camlhmp-blast-targets CLI

camlhmp included three CLI tools: camlhmp-blast-alleles, camlhmp-blast-regions, and camlhmp-blast-targets. All commands required the camlhmp schema, target FASTA file, and assembly FASTA file. camlhmp-blast-alleles aligned to target loci with alleles assigned by perfect matches. camlhmp-blast-regions aligned to large genomic regions with types assigned based on percent coverage and fewest hits. camlhmp-blast-targets aligned to individual targets with types assigned based on target matches.

The camlhmp API was grouped into the following types: engine, framework, parser, and utility. engine included modules for executing tools. framework included modules for working with the camlhmp schema. parser included modules for parsing outputs. Finally, the utility included generic modules for reading, writing, and validating data.

To demonstrate the application of camlhmp, we developed schemas for the bacterial pathogens *Pseudomonas aeruginosa*, *Streptococcus pneumoniae*, and *Staphylococcus aureus* (Table 1). These schemas can be used as stand-alone tools or as part of the Bactopia pipeline (v3.2.0) (11).

We developed camlhmp to standardize and simplify the creation and maintenance of SBT tools in microbiology. We demonstrated camlhmp with three tools: pasty, pbptyper, and sccmec. As genomic surveillance expands, tools like camlhmp are vital for sustainable typing of diverse microbial pathogens.

## Data Availability

camlhmp is available at GitHub, PyPi, and Bioconda: - https://github.com/rpetit3/camlhmp - https://pypi.org/project/camlhmp/ - https://bioconda.github.io/recipes/camlhmp/README.html camlhmp is archived on Zenodo at https://doi.org/10.5281/zenodo.19040444. Documentation for camlhmp is available at https://rpetit3.github.io/camlhmp/. camlhmp-powered Typing Tools are available from GitHub, Bioconda, and Bactopia: - pasty - https://github.com/rpetit3/pasty - pbptyper - https://github.com/rpetit3/pbptyper - sccmec - https://github.com/rpetit3/sccmec

## EDITOR'S NOTE

**Ed. Note**: This article was handled by Pat Schloss, who acted as a Guest Editor in consultation with the Editor in Chief, Irene Newton. Dr. Schloss's role as the Guest Editor of this article is outside of his duties as the chair of the Journals Committee.
